# Modulating the Properties of GPCR-Based Sensors Via C-Terminus Isoforms

**DOI:** 10.1021/acssynbio.4c00847

**Published:** 2025-04-25

**Authors:** Paola L. Marquez-Gomez, Sonia R. Damiano, Lily R. Torp, Pamela Peralta-Yahya

**Affiliations:** † School of Chemistry and Biochemistry, Georgia Institute of Technology, Atlanta, Georgia 30332, United States; ‡ School of Chemical & Biomolecular Engineering, Georgia Institute of Technology, Atlanta, Georgia 30332, United States

**Keywords:** GPCR, sensors, serotonin, yeast, isoforms

## Abstract

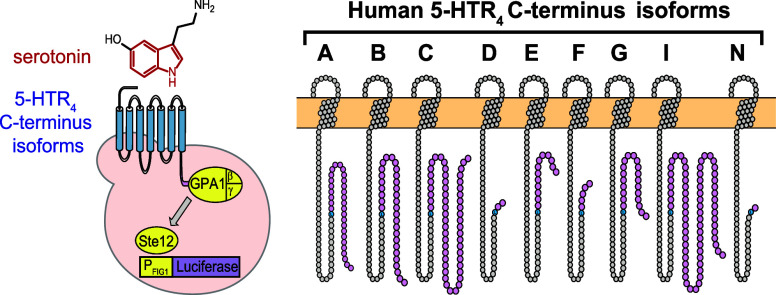

G-protein coupled receptors (GPCRs) play a key role in chemical biosensing, detecting chemicals from odorants and hormones to neurotransmitters and peptides. GPCR-based sensors in yeast can be rapidly engineered by coupling human GPCRs to the yeast mating pathway, resulting in cell fluorescence or luminescence upon chemical detection. Modulating the properties of GPCR-based sensors including their dynamic and linear ranges is nontrivial, often requiring the engineering of the yeast cell machinery. Here, we explore the use of GPCR C-terminal isoforms to modulate the properties of chemical biosensors. As a proof-of-concept, we leverage nine naturally occurring serotonin receptor 4 (5-HTR_4_) C-terminus isoforms to construct serotonin sensors with dynamic ranges spanning from 2- to 8.5-fold increases in signal after activation for a single integrated version, and from 3.4- to 62.7-fold for a double integrated version, and linear ranges reaching 5 orders of magnitude, from 10^–8^ to 10^–3^ M serotonin. Interestingly, the 5-HTR_4_ isoform-based sensors had different properties based on the chemical used to activate them, hinting at the potential differential activation of 5-HTR_4_ C-terminal isoforms in the body. Taken together, this work debuts the use of GPCR isoforms as a new strategy to rapidly modulate the dynamic and linear ranges of GPCR-based sensors in yeast.

## Introduction

G-protein coupled receptors (GPCRs) detect chemical signals on the outside of the cell and transduce this information to the inside of the cell, resulting in the regulation of genomic targets. GPCRs thus play an important role in cell signaling, regulating physiological processes including neurotransmission, growth, energy metabolism, and cardiac function.^1^ The innate ability of GPCRs to bind a wide variety of chemicals has led to the development of GPCR-based sensors in yeast. Yeast GPCR-based sensors are constructed by expressing a human GPCR on the cell surface, linking its activation to the yeast mating pathway and ultimately leading to reporter gene expression. GPCR-based sensors have wide biotechnology uses, from serving as high-throughput screening platforms to accelerate drug discovery, to being embedded in point-of-care diagnostics, to metabolic engineering applications such as quantification of microbially produced metabolites.^2^

Different GPCR-based sensor applications require different sensor properties, including dynamic and linear ranges. Although GPCR-based sensors can be quickly assembled—if the GPCR couples to the yeast machinery^3−5^—most sensors initially have limited linear and dynamic ranges, which need to be optimized before use in the desired application. To date, the modulation of GPCR-based sensor properties has largely relied on swapping the yeast G_α_-protein (GPA1) with yeast/mammalian G_α_-protein chimeras.^3,4^ In this work, we explore varying the GPCR C-terminus to achieve different sensor properties. Inspiration for this exploration is the fact that, in mammalian cells, the GPCR C-terminus alters receptor coupling to G-proteins, internalization, and membrane trafficking.^6^ By focusing only on modifications to the GPCR C-terminus, we left the chemical binding site located at the N-terminus of the receptor undisturbed.

GPCR C-terminal variations are present in nature. Fifty percent of human GPCRs undergo alternative mRNA splicing,^7^ leading to GPCR isoforms with distinct tissue distributions^6,8^ and pharmacological responses.^9,10^ For example, serotonin receptor 4 (5-HTR_4_), a pharmacological target playing roles in conditions such as irritable bowel syndrome,^11^ mood regulation, and anxiety,^12^ has ten isoforms^13^ with a wide tissue distribution, from the brain and heart to the colon and testis.^14−17^ To date, of the six 5-HTR families (5-HTR_1_, 5-HTR_2_, 5-HTR_4_, 5-HTR_5_, 5-HTR_6_, and 5-HTR_7_), yeast GPCR-based sensors for only two have been generated: 5-HTR_1_ (5-HTR_1A_,^3,18,19^ 5-HTR_1B_,^3^ 5-HTR_1D_,^18^ 5-HTR_1E_^3^) and 5-HTR_4_ (5-HTR_4B_^3−5,20−22^). The four 5-HTR_1_ isoforms studied to date have sequence variations throughout the protein, including the N-terminus, i.e., near the orthosteric binding site, and intracellular loop 3 (Supporting Information Figure S1).

Here, we explore varying the GPCR C-terminus to rapidly modulate the chemical biosensor properties. Rather than using synthetic GPCR C-termini mutants and studying their ability to modulate sensor signal, we use natural GPCR C-terminus isoforms for this endeavor as it may shed light on their roles in different human tissue. Specifically, we screened nine naturally occurring 5-HTR_4_ C-terminus isoforms to quickly optimize the dynamic and linear ranges of a serotonin sensor. To determine the extent to which the GPCR isoform-based sensor properties are ligand-dependent, we evaluated the nine sensors with two structurally dissimilar 5-HTR_4_ agonists. We find that GPCR-based sensor properties are ligand-dependent. Taken together, bioprospecting naturally occurring GPCR C-terminal isoforms is an effective strategy to rapidly optimize GPCR-based sensor properties.

## Results

### Serotonin Receptor 4 (5-HTR_4_) C-Terminus Isoforms

**Figure 1:**
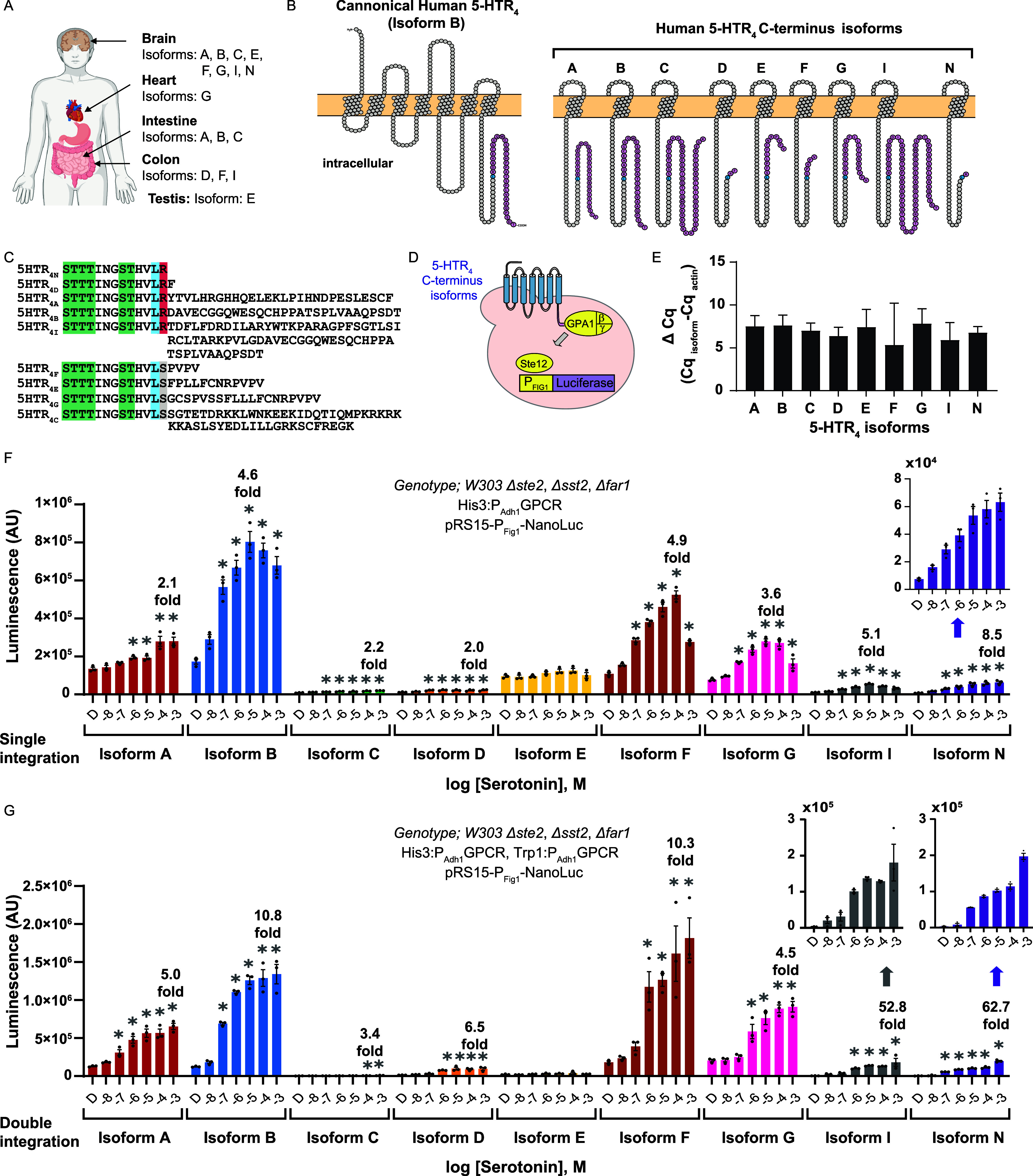
**Serotonin receptor 4 (5-HTR**_**4**_**) C-terminus isoform-based sensors**. **A.** Overview of the most common location of 5-HTR_4_ C-terminal isoforms in the body. Information obtained from.^14−17^ Figure 1a was created in Biorender. Peralta-Yahya. (2024) https://BioRender.com/j17i302. **B.** Snake plots of the nine 5-HTR_4_ C-terminal isoforms studied in this work. Snake plots were made using Protter.^27^**C.** Sequences of the C-terminus of the nine 5-HTR_4_ C-terminus isoforms. Green: S/T phosphorylation consensus for β-arrestin-dependent endocytosis. Blue: L358 the last consensus amino acid. Red: Arg^359^. Gray: Ser^359^. Sequence alignment was done using Clustal X. **D.** Schematic of 5-HTR_4_ C-terminus isoform-based sensor in yeast. Human 5-HTR_4_ isoform (blue) is expressed on the yeast cell surface. Upon serotonin binding, the receptor activates the yeast mating pathway (yellow) via the G_β/γ_ subunits, ultimately resulting in the expression of luciferase (purple). **E.** mRNA levels for each of the 5-HTR_4_ C-terminal isoforms when compared to the housekeeping gene actin (ACT1). The data was collected using the same cells used for Figure 1F. All experiments were performed in biological triplicates. The bars represent mean ± standard error of mean (SEM), *n* = 3. Data were analyzed using one-way ANOVA with Tukey’s multiple comparison using GraphPad. The ΔCt values among the isoforms were not statistically different. **F.** Dose response curves of the nine single integrated 5-HTR_4_ C-terminus isoform-based sensors in the presence of serotonin. D = DMSO, carrier solvent. Sensor strain genotype: W303 *Δste2, Δste12, Δfar1*, *His3*:P_Adh1_-5-HTR4 C-terminus isoform, and pRS415-P_Fig1_-Nanoluc. **G**. Dose response curves of the nine double integrated 5-HTR4 C-terminus isoform-based sensors in the presence of serotonin. D = DMSO, carrier solvent. Sensor strain genotype: W303 *Δste2, Δste12, Δfar1*, *His3*:P_Adh1_-5-HTR4 C-terminus isoform, *Trp1*:P_Adh1_-5-HTR4 C-terminus isoform, pRS415-P_Fig1_-Nanoluc. For **F** and **G**, all experiments were performed in biological triplicates. The bars represent mean ± standard error of mean (SEM), *n* = 3, *p ≤ 0.05. Data were analyzed using one-way ANOVA with Dunnett’s multiple comparison between DMSO and serotonin treatments using GraphPad.

Nine of the ten functional 5-HTR_4_ C-terminus isoforms have variations only at the C-terminus^16^—5-HTR_4A_, 5-HTR_4B_, 5-HTR_4C_, 5-HTR_4D_, 5-HTR_4E_, 5-HTR_4F_, 5-HTR_4G_, 5-HTR_4I_, and 5-HTR_4N_—presenting themselves as the optimal set of receptors for this study. The 5-HTR_4_ C-terminus isoforms have distinct expression in different tissue^14−17^ with isoforms A and B expressed throughout the body, isoforms E, F, G, I, and N found in the brain, isoform G found in the heart, isoform C found in the gastrointestinal tract, isoform D found in the colon, and isoform E found in the testis (Figure 1A). The nine 5-HTR_4_ C-terminus isoforms share the same sequence up to L^358^, the last consensus amino acid (Figure 1B,C, Figure S2). The Cryo-EM structure of 5-HTR_4B_ ends at C^329^; thus, no information can be gleaned about the secondary structure of the C-terminus.^23^ AlphaFold models of the 5-HTR_4_ C-terminus isoforms predict the C-terminus to be disordered except for 5-HTR_C_ and 5-HTR4_G_, in which the C-terminus forms a small intracellular helix (Figure S3).^24^

### 5-HTR_4_ C-Terminus Isoform-Based Sensor Construction – Single GPCR Integration

Previously, we engineered a yeast 5-HTR_4B_-based sensor by coupling 5-HTR_4B_ activation to the yeast G_α_ subunit, GPA1, ultimately resulting in cell luminescence.^22^ The human 5-HTR_4B_ was expressed from a strong promoter (P_Tef1_) using a multicopy plasmid (pESC), while the reporter gene was controlled by the mating pathway promoter Fig_1_ (P_Fig1_) using a single copy plasmid. Although this system resulted in up to 32-fold increase in signal after activation in the presence of serotonin, the biosensor was noisy due to expression of the GPCR from a multicopy plasmid. In this work, we reduce the biosensor noise by integrating 5-HTR_4B_, 5-HTR_4A_, 5-HTR_4C_, 5-HTR_4D_, 5-HTR_4E_, 5-HTR_4F_, 5-HTR_4G_, 5-HTR_4I_, or 5-HTR_4N_ into the yeast genome of the GPCR biosensor strain (W303 *Δste2*, *Δsst2*, *Δfar1*(25)). Briefly, the 5-HTR_4_ C-terminus isoforms were placed under the control of medium strength promoter P_ADH1_ and integrated at the *His3* locus. The luminescence reporter gene was kept under the control of P_Fig1_ as a single copy plasmid. After genome integration of the 5-HTR_4_ C-terminus isoforms, the isoforms were confirmed to be similarly expressed by performing real-time PCR. As shown in Figure 1E, the ΔCq values for the isoform are not statistically different from one another.

### Single Integrated 5-HTR_4_ C-Terminus Isoform-Based Sensor Characterization with Serotonin

Each 5-HTR_4_ C-terminus isoform has a unique profile for the signal after activation in the presence of serotonin (Figure 1F). Isoform B, the canonical 5-HTR_4_ isoform, has a linear range from 10^–7^ to 10^–5^ M achieving up to a 4.6-fold increase in signal after activation. Although Isoform B achieves the highest raw luminescence, it has a high basal sensor activity, i.e., activity in the presence of the carrier solvent, which reduces the overall dynamic range of the sensor. Isoform N, the isoform with the shortest C-terminus, has the lowest basal sensor activity, with a linear range spanning 5 orders of magnitude from 10^–8^ to 10^–3^ M serotonin and achieving up to 8.5-fold increase in signal after activation. The different linear ranges achieved with Isoform B (10^–7^ to 10^–5^ M) and Isoforms N (10^–8^ to 10^–3^ M) make them valuable for different applications. For example, if the objective is to detect serotonin in the 10^–6^ M range, the Isoform B-based sensor is preferred, as it has a linear behavior in that range. However, if the objective is to detect serotonin in the 10^–4^ M range, the Isoform N-based sensor is better suited for that application, as Isoform N has a linear behavior in that range. Isoforms A, C, and D perform similarly, achieving on average 2.1-fold increases in the signal after activation at 10^–4^ to 10^–3^ M serotonin. Interestingly, Isoform E is not activated by serotonin at any concentration. A last isoform worth highlighting is Isoform F, which has a 10^–7^ to 10^–4^ linear range and achieves a 4.9-fold increase in signal intensity after activation at 10^–4^ M serotonin.

### Double Integrated 5-HTR_4_ C-Terminus Isoform-Based Sensor Construction and Characterization

In the context of yeast GPCR-based sensors, maximum signal increase after activation has been observed when two copies of the GPCR are integrated in the genome.^26^ Interested in determining if a second integration of the 5-HTR_4_ C-terminus isoforms would increase the sensors’ signal after activation, we introduced a second copy of each isoform also under control of the P_ADH1_ at the *Trp1* locus. As shown in Figure 1G, the signal after activation of all doubly integrated 5-HTR_4_ C-terminus isoform-based sensors increases. Double integration of Isoform B or Isoform F results in an ∼10-fold increase in signal after activation. Most remarkable, however, is the performance of Isoform I and Isoform N that jump to 52.8-fold and 62.7-fold increases in signal after activation, respectively. Pivotal to the sensor performance improvement seen for Isoforms I and N is their maintenance of very low basal sensor activation.

### Characterization of the Singly Integrated 5-HTR_4_ C-Terminus Isoform-Based Sensors with Known Serotonin Agonists

**Figure 2:**
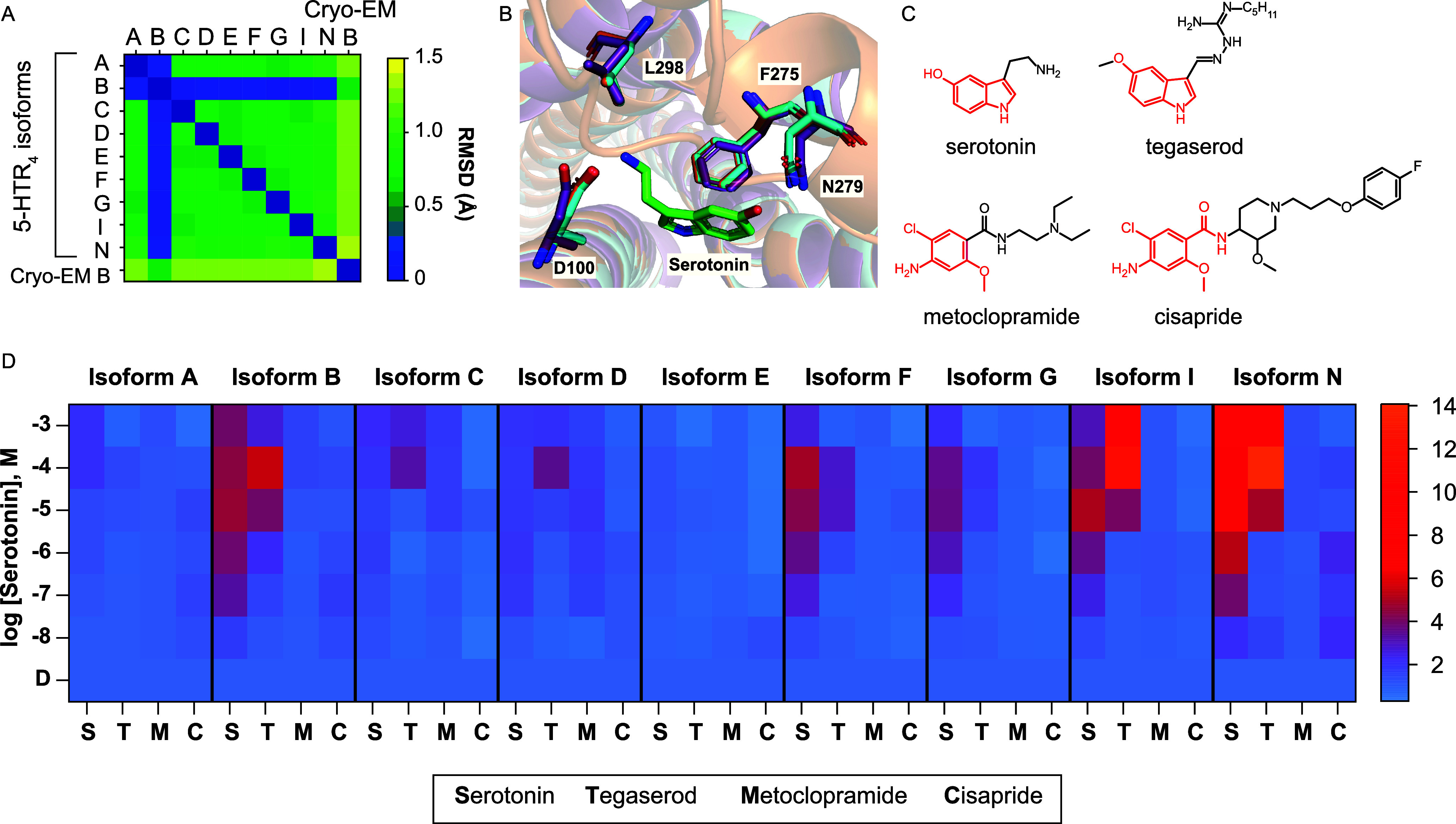
**Ligand influence on 5-HTR**_**4**_**C-terminus isoform-based sensor properties. A**. Heat map of the root-mean-square deviation (RMSD) of the active site of the AlphaFold models of the nine 5-HTR_4_ C-terminus isoforms and the Cryo-EM structure of 5-HTR_4B_ (PDB entry 7XT9). **B**. Structural alignment of the 5-HTR_4B_ Cryo-EM structure (PDB: 7XT9, purple), and AlphaFold models of 5-HTR_4B_ (cyan) and 5-HTR_4G_ (orange). **C**. Structural comparison of serotonin and the three ligands tested: tegaserod, which shares the 5-hydroxyindole moiety with serotonin and metoclopramide, and cisapride, which relies on a hydroxyaniline moiety. **D**. Fold increase in signal after activation of single integrated 5-HTR_4_ C-terminus isoform-based sensor with serotonin (S), tegaserod (T), metoclopramide (M), and cisapride (C). All experiments were performed in biological triplicates. Raw data can be found in Figures S4 & S5.

Given the nine 5-HTR_4_ C-terminus isoforms expressed at similar levels (Figure 1E) and that the chemical binding site of the isoforms are almost identical (Figure 2A,B)—<1 Å RMSD across the active sites of the AlphaFold modeled isoforms—we hypothesized that the system could be used to evaluate how structurally diverse 5-HTR_4_ ligands (Figure 2C) affect GPA1 coupling and ultimate sensor signaling. It is well-established that GPCR signaling response can be modulated by the ligand itself, which induces rearrangement of the transmembrane helices^28^ resulting in different positions of the intracellular loops and C-terminus interacting with the G-protein, ultimately affecting signaling. For example, tegaserod acts as a 5-HTR_4_ agonist, while piboserod acts as an antagonist. In the context of 5-HTR_4_ C-terminal isoforms, there is evidence that they respond differently to the same ligand. In mammalian CHO cells, renzapride is almost 20 times more potent at activating 5-HTR_4D_ than 5-HTR_4G_ measured as cAMP accumulation.^29^

We evaluated the properties of 5-HTR_4_ C-terminus isoform-based sensors with three structurally different agonists: tegaserod, metoclopramide, and cisapride (Figure 2C). Structurally, tegaserod shares the 5-hydroxyindole moiety with serotonin, while metoclopramide and cisapride use a hydroxyaniline moiety to provide the necessary hydrophobicity. Figure 2D (Figures S4 & S5) shows the x-fold increase in signal after activation of the nine 5-HTR4 C-terminus isoform-based sensors with serotonin, tegaserod, metoclopramide, and cisapride. We observe that the sensors result in different fold activations when activated by different compounds despite having an almost identical active site. For example, the canonical Isoform B is activated by both serotonin (4.6-fold) and tegaserod (5.5-fold) which share the 5-hydroxyindole moiety, but not by metoclopramide or cisapride that share a hydroxyaniline moiety. Interestingly, Isoform I is more strongly activated by tegaserod (11.6-fold) than serotonin (5.6-fold). A similar trend is seen with Isoform N, where tegaserod achieves a 14.1-fold increase while serotonin achieves 8.9-fold.

## Conclusion

In this work, we evaluated the utility of GPCR C-terminal isoforms to rapidly modulate the properties of a chemical biosensor. We find that bioprospecting naturally occurring GPCR-based C-terminal isoforms is a successful strategy in expanding both the linear and dynamic ranges of GPCR-based sensors. Taken together, serotonin sensors spanning from 2–8.5-fold increase in signal after activation for a single integrated version, and from 3.4–62.7-fold for a double integrated version, and linear ranges reaching up to 5 orders of magnitude—10^–8^ to 10^–3^ M serotonin—were generated. This suite of 5-HTR_4_ C-terminus isoform-based sensors now allows picking of serotonin sensors for different applications. For example, if detection of 10^–6^ M serotonin is desired, the Isoform B-based sensor would be the best choice, as it has a linear range around that concentration. On the other hand, if detection of 10^–4^ M serotonin is desired, an isoform N-based sensor would be the preferred one as it has a linear range around that concentration. We also find that the double integration of 5-HTR_4_-based sensors in the chromosome significantly increases the sensor signal over that seen in the single integrated sensor versions. The low basal activity of Isoform I and Isoform N allows them to achieve more than a 50-fold increase in signal after activation.

Interestingly, GPCR isoform-based sensors have different properties depending on the ligands used. The analysis performed with three known 5-HTR_4_ agonists, tegaserod, metoclopramide, and cisapride, shows how isoforms are differentially activated by these ligands, hinting at GPCR isoforms potentially being differentially activated throughout the body by different ligands.

Placing this work in the broader biosensor context, unlike allosteric transcription factor-based sensors in which the sensor’s features can only be modulated by engineering the ligand binding domain or altering the DNA operator sequence,^2^ GPCR-based sensors can be additionally modulated by changing their GPCR C-terminus. Improved coupling of human GPCR to the yeast machinery likely increases the active concentration of the mating pathway transcription factor Ste12, resulting in a higher level of reporter gene transcription. Finally, changes to the GPCR C-terminus should work synergistically with changes to other parts of the system toward enhancing the sensor’s properties, including improving the GPCR–ligand interactions and the transcription factor/promoter affinity.

## Materials and Methods

### Materials

Serotonin hydrochloride (S0370) was purchased from TCI chemicals. Metoclopramide hydrochloride (M0763) and Tegaserod maleate (SML1504) were purchased from Sigma-Aldrich. Nano-Glo Luciferase Assay System (N1120) was purchased from Promega.

### 5-HTR_4_ C-Terminus Isoforms Plasmid Construction (Multicopy Plasmids)

The 5-HTR_4B_ sensor (PPY2360) was previously generated.^22^ The eight remaining 5-HTR_4_ C-terminus isoforms were codon-optimized for *Saccharomyces cerevisiae*, commercially synthesized (ThermoFisher) and cloned into pESC-His3-P_TEF_ (pKM111)^25^ at *Bam*HI/*Sac*II via Gibson assembly to generate pESC-His3-P_TEF1_-5-HTR_4A_ (pPM58), pESC-His3-P_TEF1_-5-HTR_4C_ (pPM62), pESC-His3-P_TEF1_-5-HTR_4D_ (pPM63), pESC-His3-P_TEF1_-5-HTR_4E_ (pPM61), pESC-His3-P_TEF1_-5-HTR_4F_ (pPM60), pESC-His3-P_TEF1_-5-HTR_4G_ (pPM59), pESC-HIS3-P_TEF1_-5-HTR_4I_ (pLT3), pESC-HIS3-P_TEF1_-5-HTR_4N_ (pLT4). Constructs were sequence-verified using primers LT62/LT63.

### 5-HTR_4_ C-Terminus Isoforms Plasmid Construction (Integration Plasmids)

For the single integrated 5-HTR_4_ C-terminus isoform-based sensors, the 5-HTR_4_ C-terminus isoforms were amplified from their respective pESC vectors using primers that introduced an FLAG tag at the N-terminus. The FLAG-tagged 5-HTR_4_ C-terminus isoforms were introduced into pNH603-*C. glabrata* His3-P_ADH1_-MCP-VP64- *C. albicans* T_ADH1_ (pJZC522^30^) between *NotI*/*XhoI* (replacing MCP-VP64) via Gibson assembly to generate pInt-His-P_ADH1_-FLAG-5-HTR_4A_ (pPM136), pInt-His-P_ADH1_-FLAG-5-HTR_4B_ (pPM137), pInt-His-P_ADH1_-FLAG-5-HTR_4C_ (pPM138), pInt-His-P_ADH1_-FLAG-5-HTR_4D_ (pPM139), pInt-His3-P_ADH1_-FLAG-5-HTR_4E_ (pPM140), pInt-His-P_ADH1_-FLAG-5-HTR_4F_ (pPM141), pInt-His-P_ADH1_-FLAG-5-HTR_4G_ (pPM142), pInt-His-P_ADH1_-FLAG-5-HTR_4I_ (pPM143), and pInt-His-P_ADH1_-FLAG-5-HTR_4N_ (pPM144). Constructs were sequence-verified by using whole plasmid sequencing. To generate the integration plasmids for the double integrated 5-HTR_4_ C-terminus isoform-based sensors, the P_ADH1-_FLAG-5-HTR_4_ C-terminus isoforms were amplified from their respective pInt-His vectors and introduced into pJZC530-derived^31^ pIntTrp-P_TEF1_-GPA1–5AA-G_s_-*C. albicans* T_ADH1_ (pPM189) between *ApaI/BamHI* (replacing P_TEF1_-GPA1–5AA-G_s_) via Gibson assembly to generate pInt-Trp-P_ADH1_-FLAG-5-HTR_4A_ (pPM289), pInt-Trp-P_ADH1_-FLAG-5-HTR_4B_ (pPM290), pInt-Trp-P_ADH1_-FLAG-5-HTR_4C_ (pPM291), pInt-Trp-P_ADH1_-FLAG-5-HTR_4D_ (pPM292), pInt-Trp-P_ADH1_-FLAG-5-HTR_4E_ (pPM293), pInt-Trp-P_ADH1_-FLAG-5-HTR_4F_ (pPM294), pInt-Trp-P_ADH1_-FLAG-5-HTR_4G_ (pPM295), pInt-Trp-P_ADH1_-FLAG-5-HTR_4I_ (pPM296), and pInt-Trp-P_ADH1_-FLAG-5-HTR_4N_ (pPM297). Constructs were sequence-verified using whole plasmid sequencing.

### Single Integrated 5-HTR_4_ C-Terminus Isoform-Based Sensor Strain Construction

pPM136, pPM137, pPM138, pPM139, pPM140, pPM141, pPM142, pPM143, and pPM144 were linearized by digesting with *PmeI* and transforming the linear DNA into PPY140 (*S. cerevisiae* W303 *leu2–3,112 trp1–1 can1–100 ura3–1 ade2–1 his3–11,15 Δfar1, Δste2, Δsst2*).^25^ Successful yeast integrations were selected on media lacking histidine. GPCR integrations were verified via PCR using primers PB140/PB141. The single integrated 5-HTR_4_ C-terminus strains PPY2753 (5-HTR_4A_), PPY2754 (5-HTR_4B_), PPY2755 (5-HTR_4C_), PPY2756 (5-HTR_4D_), PPY2763 (5-HTR_4E_), PPY2757 (5-HTR_4F_), PPY2758 (5-HTR_4G_), PPY2759 (5-HTR_4I_), and PPY2760 (5-HTR_4N_) were transformed with pRS415-Leu2-P_Fig1_-NanoLuc^22^ to generate sensors for 5-HTR_4A_ (PPY2796), 5-HTR_4B_ (PPY2797), 5-HTR_4C_ (PPY2798), 5-HTR_4D_ (PPY2799), 5-HTR_4E_ (PPY2800), 5-HTR_4F_ (PPY2801), 5-HTR_4G_ (PPY2802), 5-HTR_4I_ (PPY2803), and 5-HTR_4N_ (PPY2804). The no receptor control (PPY2805), i.e., the GPCR-based sensors strain carrying no integrated receptor, was generated by transforming pRS415-Leu2-P_Fig1_-NanoLuc^22^ into PPY140.

### Double Integrated 5-HTR_4_ C-Terminus Isoform-Based Sensor Strain Construction

pPM289, pPM290, pPM291, pPM292, pPM293, pPM294, pPM295, pPM296, and pPM297 were linearized by digesting with *PmeI* and transforming the linear DNA into PPY2753 (5-HTR_4A_), PPY2754 (5-HTR_4B_), PPY2755 (5-HTR_4C_), PPY2756 (5-HTR_4D_), PPY2763 (5-HTR_4E_), PPY2757 (5-HTR_4F_), PPY2758 (5-HTR_4G_), PPY2759 (5-HTR_4I_), and PPY2760 (5-HTR_4N_). Successful yeast integrations were selected on media lacking histidine and tryptophan. GPCR integrations were verified via PCR using primers PB140/PB142. PPY3533 (5-HTR_4A_), PPY3534 (5-HTR_4B_), PPY3535 (5-HTR_4C_), PPY3536 (5-HTR_4D_), PPY3537 (5-HTR_4E_), PPY3538 (5-HTR_4F_), PPY3539 (5-HTR_4G_), PPY3540 (5-HTR_4I_), and PPY3541 (5-HTR_4N_) were transformed with pRS415-Leu2-P_Fig1_-NanoLuc to generate sensors for 5-HTR_4A_ (PPY3598), 5-HTR_4B_ (PPY3599), 5-HTR_4C_ (PPY3600), 5-HTR_4D_ (PPY3601), 5-HTR_4E_ (PPY3602), 5-HTR_4F_ (PPY3603), 5-HTR_4G_ (PPY3604), 5-HTR_4I_ (PPY3605), and 5-HTR_4N_ (PPY3606).

### 5-HTR_4_ C-Terminus Isoform-Based Sensor Activation

#### Single integrated 5-HTR

_*4*_*C-terminus isoform-based sensors.* Overnight cultures for three independent colonies of each PPY2796, PPY2797, PPY2798, PPY2799, PPY2800, PPY2801, PPY2802, PPY2803, or PPY2804 were used to inoculate 50 mL of synthetic complete medium with 2% glucose lacking histidine and leucine (SD(HL^–^)) to an OD_600_ = 0.06. After 18 h at 15 °C (150 rpm), the cultures were centrifuged (3500 rpm, 10 min) and resuspended in SD(HL^–^) to an OD_600_ = 1. In a white, flat-bottomed 96-well plate, 190 μL pH = 7 SD (HL^–^), 8 μL of cells, and 2 μL of serotonin (final concentration 10^–9^ to 10^–3^ M), or DMSO as a control were added. After chemical incubation (2.5 h, 30 °C, 250 rpm), 20 μL of 1:100 mixture of NanoLuc substrate to NanoLuc buffer was added, and the reaction incubated for 30 min (30 °C, 250 rpm). Luminescence was read in a Biotek Synergy 2 instrument using default settings. The same protocol was followed to detect tegaserod, metoclopramide, and cisapride. *Double integrated 5-HTR*_4_*C-terminus isoform-based sensors.* Overnight cultures for three independent colonies of each PPY3598, PPY3599, PPY3600, PPY3601, PPY3602, PPY3603, PPY3604, PPY3605, or PPY3606 were used to inoculate 5 mL of synthetic complete medium with 2% glucose lacking histidine, tryptophan, and leucine (SD(HWL^–^)) to an OD_600_ = 0.6. After 18 h at 15 °C (150 rpm), the cultures were centrifuged (3500 rpm, 10 min) and resuspended in SD(HWL^–^) to an OD_600_ = 1. In a white, flat-bottomed 96-well plate, 190 μL pH = 7 SD (HWL^–^), 8 μL of cells, and 2 μL of serotonin (final concentration 10^–9^ to 10^–3^ M), or DMSO as a control were added. After chemical incubation (2.5 h, 30 °C, 250 rpm), 20 μL of 1:100 mixture of NanoLuc substrate to NanoLuc buffer were added, and the reaction incubated for 30 min (30 °C, 250 rpm). Luminescence was read in a Biotek Synergy 2 using default settings.

### mRNA Quantification

Overnight cultures for three independent colonies of PPY2796, PPY2797, PPY2798, PPY2799, PPY2800, PPY2801, PPY2802, PPY2803, or PPY2804 were used to inoculate 50 mL of SD(HL^–^) to an OD _600_ = 0.06. After 18 h at 15 °C (150 rpm), the cultures were centrifuged (3500 rpm, 10 min) and resuspended in SD(HL^–^) to an OD_600_ = 1. One mL of cells at OD = 1 were pelleted, and total RNA was extracted using RNeasy Mini kit (Qiagen). RNA concentrations were measured using a Nanodrop Lite spectrophotometer. Reverse transcription was done using 1000 ng of total RNA using QuantiTect Reverse Transcription kit (Qiagen). Real-time PCR reactions were set up using the QuantiTect SYBR Green PCR kit (Qiagen) using 3 μL of 250 ng of cDNA and read using an Applied Biosciences StepOnePlus Real-Time PCR system. Reactions were set up as triplicates using primers PM76/PM77 for all isoforms and ACT-F/ACT-R for actin. 5-HTR_4_ C-terminus isoform expression was normalized to the housekeeping gene ACT1 and were compared using comparative Cq method using the equation below:
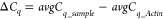


### AlphaFold Isoform Structures

The structure of 5-HTR_4B_ was obtained from the AlphaFold web server (Q13639).^24^ The structures of 5-HTR_4_ isoforms A, C, D, E, F, G, I, and N were obtained using the monomer model in AlphaFold Colab.

### RMSD Calculations

The cryo-EM structure, the 5-HTR_4B_-Gs complex (PDB: 7XT9), and AlphaFold structures of 5-HTR_4_: A, B, C, D, E, F, G, I, and N were entered into PyMOL. To determine the binding pocket, residues 5 Å from serotonin in the cryo-EM structure and AlphaFold structures were selected. PyMOL was used to obtain the RMSD value for the binding pocket.

## Supporting Information Available

**Supporting Table 1**. Table of Plasmids. **Supporting Table 2**. Table of Strains. **Supporting Table 3**. Table of Primers. **Figure S1**. Sequence alignment of 5-HTR_1_ isoforms studied to date. **Figure S2**. Sequence alignment of 5-HTR_4_ C-terminus isoforms studied in this work. **Figure S3**. AlphaFold structural predictions for 5-HTR4 C-terminus isoforms A, B, C, D, E, F, G, I and N. **Figure S4**. Dose response curves of 5-HTR_4_ C-terminus isoform-based sensors with serotonin, tegaserod, metoclopramide and cisapride. **Figure S5**. Data on fold increase in signal after activation of single integrated 5-HTR4 C-terminus isoform-based sensor with serotonin (S), tegaserod (T), metoclopramide (M), and cisapride (C). **Sequences. References** (PDF)

## Abbreviations

GPCR: G protein-coupled receptor
5-HTR_4_: Serotonin receptor 4
