# Supplementary material for: Modulating the Properties of GPCR-Based Sensors Via C-Terminus Isoforms
Source: ACS Synth Biol. 2025 Apr 25;14(5):1853–60. doi: 10.1021/acssynbio.4c00847 (PMC12090342; doi:10.1021/acssynbio.4c00847)
Supplement: Supplementary file 1 — sb4c00847_si_001.pdf [file sb4c00847_si_001.pdf]

Modulating the properties of GPCR-based sensors via C-terminus isoforms

Paola L. Marquez-Gomez[1], Sonia Damiano[1], Lily R. Torp[1], Pamela Peralta-Yahya[1,2*]

[1] School of Chemistry and Biochemistry, Georgia Institute of Technology, Atlanta, GA 30332

[2] School of Chemical & Biomolecular Engineering, Georgia Institute of Technology, Atlanta, GA 30332

* Corresponding author

Email: pperalta-yahya@chemistry.gatech.edu

Supplementary information

Supporting Table 1. Table of Plasmids S2

Supporting Table 2. Table of Strains S3

Supporting Table 3. Table of Primers S4

Figure S1. Sequence alignment of 5-HTR$_1$ isoforms studied to date S6

Figure S2. Sequence alignment of 5-HTR$_4$ C-terminus isoforms studied in this work S7

Figure S3. AlphaFold structural predictions for 5-HTR$_4$ C-terminus isoforms A, B, C, D, E, F, G, I and N S8

Figure S4. Dose response curves of 5-HTR$_4$ C-terminus isoform-based sensors with serotonin, tegaserod, metoclopramide and cisapride S9

Figure S5. Data on fold increase in signal after activation of single integrated 5-HTR$_4$ C-terminus isoform-based sensor with serotonin (S), tegaserod (T), metoclopramide (M) and cisapride (C). S10

Sequences S12

References S13

Supporting Table 1. Table of Plasmids

Plasmid Number	Plasmid Name	Description	Citation
PPY111	pKM111	pESC-His3-P_{TEF}	[1]
PPY1192	pTMC18	pESC-His3-P_{TEF1}-5-HTR_{4B}	[2]
PPY1740	pEY15	pRS415-Leu2-P_{FIG1}-NanoLuc	[3]
PPY2161	pJZC522	pNH603-*C. glabrata* His3-P_{ADH1}-MCP-VP64-*C. albicans* T_{ADH1}	[4]
PPY2162	pJZC530	pNH604-*C. glabrata* Trp1-P_{TetO1x}-Venus- *C. albicans* T_{ADH1}	[5]
PPY2084	pLT3	pESC-HIS3-P_{TEF1}-5-HTR_{4I}	This work
PPY2085	pLT4	pESC-HIS3-P_{TEF1}-5-HTR_{4N}	This work
PPY2378	pPM58	pESC-His3-P_{TEF1}-5-HTR_{4A}	This work
PPY2380	pPM59	pESC-His3-P_{TEF1}-5-HTR_{4G}	This work
PPY2381	pPM61	pESC-His3-P_{TEF1}-5-HTR_{4E}	This work
PPY2382	pPM62	pESC-His3-P_{TEF1}-5-HTR_{4C}	This work
PPY2383	pPM63	pESC-His3-P_{TEF1}-5-HTR_{4D}	This work
PPY2395	pPM60	pESC-His3-P_{TEF1}-5-HTR_{4F}	This work
PPY2734	pPM136	pInt-His-P_{ADH1}-FLAG-5-HTR_{4A}-*CA* T_{ADH1}	This work
PPY2735	pPM137	pInt-His-P_{ADH1}-FLAG-5-HTR_{4B}-*CA* T_{ADH1}	This work
PPY2736	pPM138	pInt-His-P_{ADH1}-FLAG-5-HTR_{4C}-*CA* T_{ADH1}	This work
PPY2737	pPM139	pInt-His-P_{ADH1}- FLAG-5-HTR_{4D}-*CA* T_{ADH1}	This work
PPY2738	pPM140	pInt-His-P_{ADH1}-FLAG-5-HTR_{4E}-*CA* T_{ADH1}	This work
PPY2739	pPM141	pInt-His-P_{ADH1}-FLAG-5-HTR_{4F}-*CA* T_{ADH1}	This work
PPY2740	pPM142	pInt-His-P_{ADH1}-FLAG-5-HTR_{4G}-*CA* T_{ADH1}	This work
PPY2741	pPM143	pInt-His-P_{ADH1}-FLAG-5-HTR_{4I}-*CA* T_{ADH1}	This work
PPY2742	pPM144	pInt-His-P_{ADH1}-FLAG-5-HTR_{4N}-*CA* T_{ADH1}	This work
PPY2937	pPM189	pInt-Trp-P_{TEF1}-GPA1-5AA-G_s-*CA* T_{ADH1}	This work
PPY3480	pPM289	pInt-Trp-P_{ADH1}-FLAG-5-HTR_{4A}-*CA* T_{ADH1}	This work
PPY3481	pPM290	pInt-Trp-P_{ADH1}-FLAG-5-HTR_{4B}-*CA* T_{ADH1}	This work
PPY3482	pPM291	pInt-Trp-P_{ADH1}-FLAG-5-HTR_{4C}-*CA* T_{ADH1}	This work
PPY3483	pPM292	pInt-Trp-P_{ADH1}-FLAG-5-HTR_{4D}-*CA* T_{ADH1}	This work
PPY3484	pPM293	pInt-Trp-P_{ADH1}-FLAG-5-HTR_{4E}-*CA* T_{ADH1}	This work

PPY3485	pPM294	pInt-Trp-P_{ADH1}-FLAG-5-HTR_{4F}-*CA* T_{ADH1}	This work
PPY3486	pPM295	pInt-Trp-P_{ADH1}-FLAG-5-HTR_{4G}-*CA* T_{ADH1}	This work
PPY3487	pPM296	pInt-Trp-P_{ADH1}-FLAG-5-HTR_{4I}-*CA* T_{ADH1}	This work
PPY3488	pPM297	pInt-Trp-P_{ADH1}-FLAG-5-HTR_{4N}-*CA* T_{ADH1}	This work

Supporting Table 2: Table of Strains

**Strain Number**	**Description**	**Citation**
PPY140	*S. cerevisiae* W303MATa *ade2-1 ura3-1 his3-11 trp1-1 leu2-3 leu2-112 can1-100* Δ*far1* Δ*ste2* Δ*sst2*	ATCC
PPY2753	PPY140 *His3:P_{ADH1}-FLAG-5-HTR_{4A}-CA T_{ADH1}*	This work
PPY2754	PPY140 *His3:P_{ADH1}-FLAG-5-HTR_{4B}-CA T_{ADH1}*	This work
PPY2755	PPY140 *His3:P_{ADH1}-FLAG-5-HTR_{4C}-CA T_{ADH1}*	This work
PPY2756	PPY140 *His3:P_{ADH1}-FLAG-5-HTR_{4D}-CA T_{ADH1}*	This work
PPY2763	PPY140 *His3:P_{ADH1}-FLAG-5-HTR_{4E}-CA T_{ADH1}*	This work
PPY2757	PPY140 *His3:P_{ADH1}-FLAG-5-HTR_{4F}-CA T_{ADH1}*	This work
PPY2758	PPY140 *His3:P_{ADH1}-FLAG-5-HTR_{4G}-CA T_{ADH1}*	This work
PPY2759	PPY140 *His3:P_{ADH1}-FLAG-5-HTR_{4I}-CA T_{ADH1}*	This work
PPY2760	PPY140 His3:P_{ADH1}-FLAG-5-HTR_{4N}-*CA* T_{ADH1}	This work
PPY2796	PPY2753, pRS415-Leu2-P_{FIG1}-NanoLuc	This work
PPY2797	PPY2754, pRS415-Leu2-P_{FIG1}-NanoLuc	This work
PPY2798	PPY2755, pRS415-Leu2-P_{FIG1}-NanoLuc	This work
PPY2799	PPY2756, pRS415-Leu2-P_{FIG1}-NanoLuc	This work
PPY2800	PPY2763, pRS415-Leu2-P_{FIG1}-NanoLuc	This work
PPY2801	PPY2757, pRS415-Leu2-P_{FIG1}-NanoLuc	This work
PPY2802	PPY2758, pRS415-Leu2-P_{FIG1}-NanoLuc	This work
PPY2803	PPY2759, pRS415-Leu2-P_{FIG1}-NanoLuc	This work
PPY2804	PPY2760, pRS415-Leu2-P_{FIG1}-NanoLuc	This work
PPY2805	PPY140, pRS415-Leu2-P_{FIG1}-NanoLuc	This work
PPY3533	PPY2753 *Trp1:P_{ADH1}-FLAG-5-HTR_{4A}-CA T_{ADH1}*	This work
PPY3534	PPY2754 *Trp1:P_{ADH1}-FLAG-5-HTR_{4B}-CA T_{ADH1}*	This work
PPY3535	PPY2755 *Trp1:P_{ADH1}-FLAG-5-HTR_{4C}-CA T_{ADH1}*	This work

PPY3536	PPY2756 *Trp1:*P_{ADH1}*-FLAG-5-HTR*$_{4D}$*-CA* T_{ADH1}	This work
PPY3537	PPY2763 *Trp1:*P_{ADH1}*-FLAG-5-HTR*$_{4E}$*-CA* T_{ADH1}	This work
PPY3538	PPY2757 *Trp1:*P_{ADH1}*-FLAG-5-HTR*$_{4F}$*-CA* T_{ADH1}	This work
PPY3539	PPY2758 *Trp1:*P_{ADH1}*-FLAG-5-HTR*$_{4G}$*-CA* T_{ADH1}	This work
PPY3540	PPY2759 *Trp1:*P_{ADH1}*-FLAG-5-HTR*$_{4I}$*-CA* T_{ADH1}	This work
PPY3541	PPY2760 *Trp1:*P_{ADH1}*-FLAG-5-HTR*$_{4N}$*-CA* T_{ADH1}	This work
PPY3598	PPY3533, pRS415-Leu2-P_{FIG1}-NanoLuc	This work
PPY3599	PPY3534, pRS415-Leu2-P_{FIG1}-NanoLuc	This work
PPY3600	PPY3535, pRS415-Leu2-P_{FIG1}-NanoLuc	This work
PPY3601	PPY3536, pRS415-Leu2-P_{FIG1}-NanoLuc	This work
PPY3602	PPY3537, pRS415-Leu2-P_{FIG1}-NanoLuc	This work
PPY3603	PPY3538, pRS415-Leu2-P_{FIG1}-NanoLuc	This work
PPY3604	PPY3539, pRS415-Leu2-P_{FIG1}-NanoLuc	This work
PPY3605	PPY3540, pRS415-Leu2-P_{FIG1}-NanoLuc	This work
PPY3606	PPY3541, pRS415-Leu2-P_{FIG1}-NanoLuc	This work

Supporting Table 3: Table of Primers

**Primer name**	**Sequence**
PM76	agttggatgctaacgtttct
PM77	tctatcccaacaaacagcaa
PM86	tcaactatctcatatacaatctctctcgagatggactacaaagacgacgacgacaaaggctcaggcga taagttggatgctaacgtttct
PM87	gcttagagctccaccgcggtggcggccgctcagaagcatgattccaggga
PM88	tgcttagagctccaccgcggtggcggccgcttaggtatcagatggttgagctg
PM89	tgcttagagctccaccgcggtggcggccgcctattttccttccctaaaacatgac
PM90	tgcttagagctccaccgcggtggcggccgcttagaatctcaagacatgtgtggaac
PM91	tgcttagagctccaccgcggtggcggccgcttagacaggaactggtctattgca
PM92	tgcttagagctccaccgcggtggcggccgcttagacaggaactggactcaagaca
PM93	tgcttagagctccaccgcggtggcggccgcttagacaggaactggtctattgca
PM94	tgcttagagctccaccgcggtggcggccgcttaggtatcgctcggctg
PM95	tgcttagagctccaccgcggtggcggccgcttatctcaagacatgtgtggaacc
PM125	catagggataaaatgtgataactaatcagcggtaccgggccctaaaac

PM126	catgttgcaggtgtctagaactagtggatcctcagaagcatgattccaggga
PM127	catgttgcaggtgtctagaactagtggatccttaggtatcagatggttgagctg
PM128	catgttgcaggtgtctagaactagtggatccctattttccttccctaaaacatgac
PM129	catgttgcaggtgtctagaactagtggatccttagaatctcaagacatgtgtggaac
PM130	catgttgcaggtgtctagaactagtggatccttagacaggaactggtctattgc
PM131	catgttgcaggtgtctagaactagtggatccttagacaggaactggactcaaga
PM133	catgttgcaggtgtctagaactagtggatccttaggtatcgctcggctg
PM134	catgttgcaggtgtctagaactagtggatccttatctcaagacatgtgtggaacc
PB140	cccccttttgcttataattgtgtgg
PB141	accaccagaacggccgttagatc
PB142	aaaagttcacctgtcccacctgc
ACT-F	ttctgaggttgctgctttgg
ACT-R	accgacgatagatgggaagac

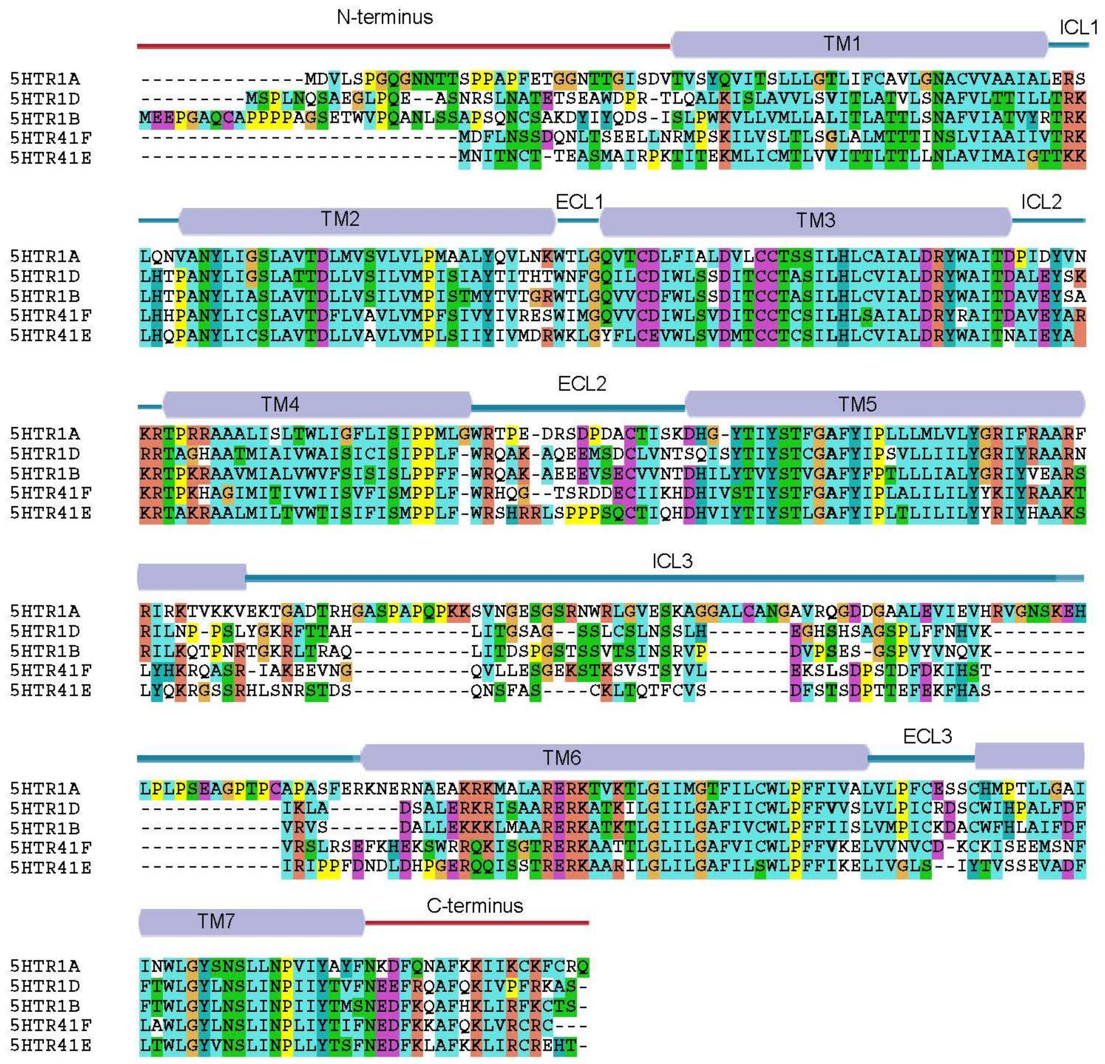

Figure S1. Sequence alignment of 5-HT$_1$ isoforms studied to date[6-8]. N-term: N-terminus. TM: Transmembrane domain. ICL: Intracellular Loop. ECL: Extracellular Loop. C-term; C-terminus. Sequence alignment done using Clustal X. Secondary structure identification obtained from GPCRdb (https://gpcrdb.org/).

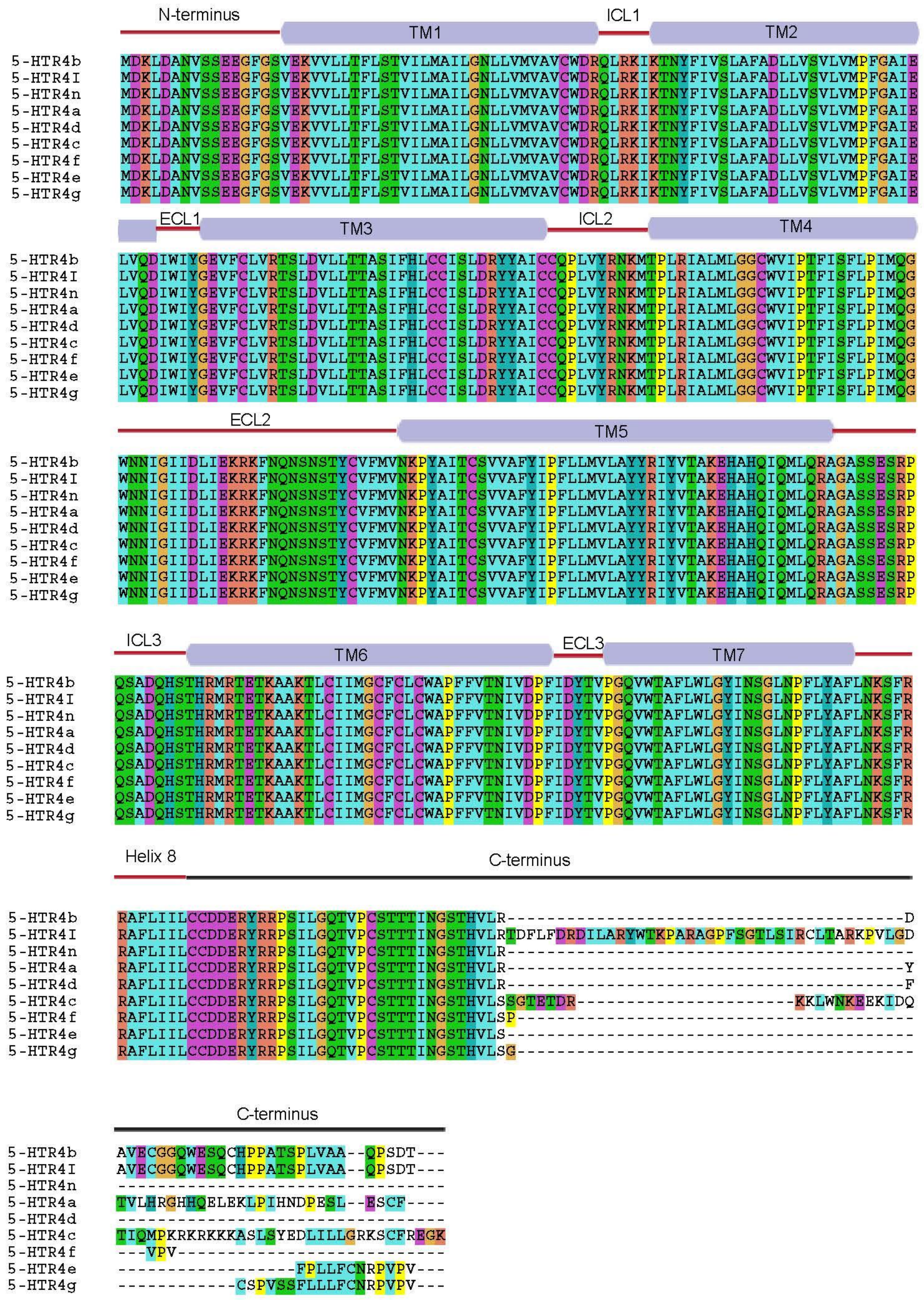

Figure S2. Sequence alignment of 5-HTR$_4$ C-terminus isoforms studied in this work. N-term: N-terminus. TM: Transmembrane domain. ICL: Intracellular Loop. ECL: Extracellular Loop. C-term; C-terminus. Sequence alignment done using Clustal X. Secondary structure identification obtained from GPCRdb (https://gpcrdb.org/).

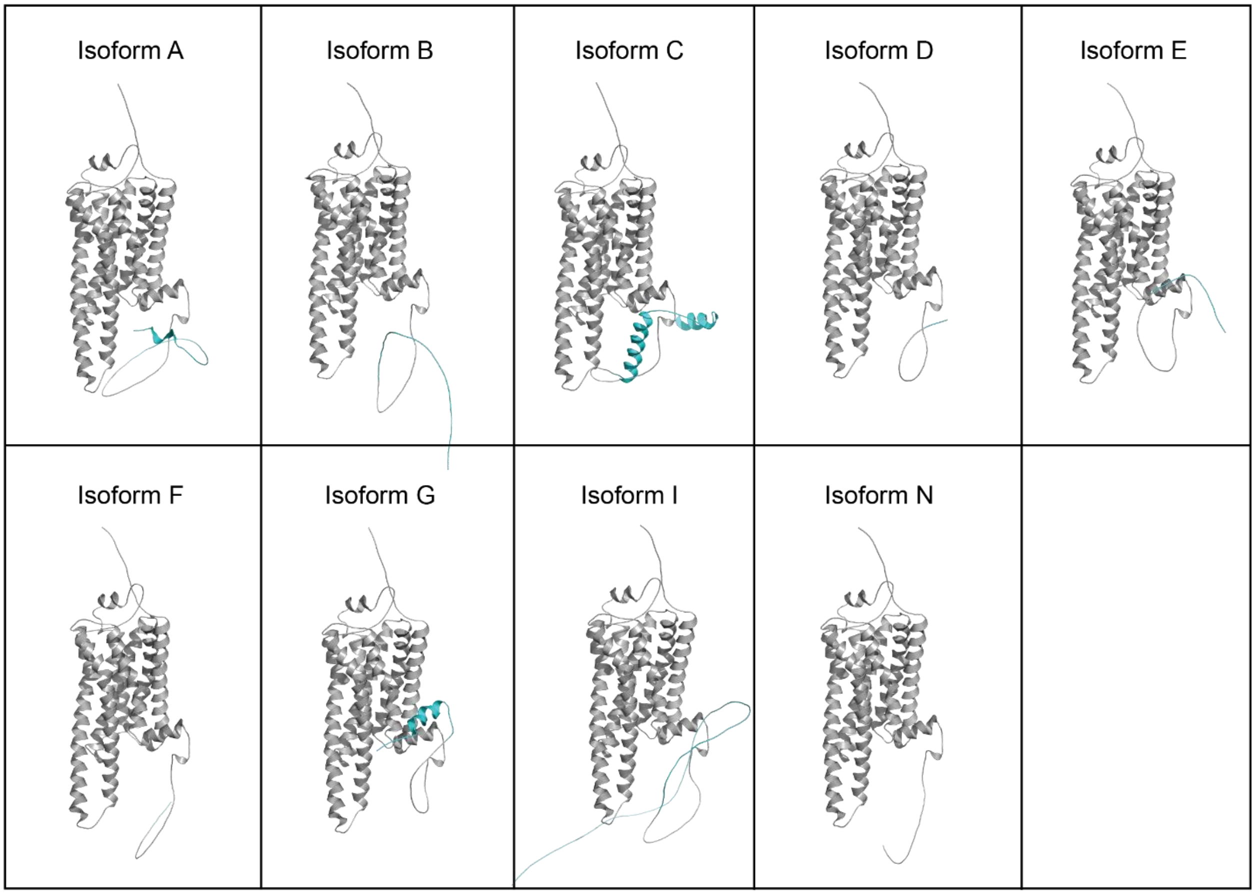

Figure S3. AlphaFold structural predictions for 5-HTR$_4$ C-terminus isoform. Structures visualized using PyMOL. Structures in grey. C-terminus starting at L^{358} in cyan.

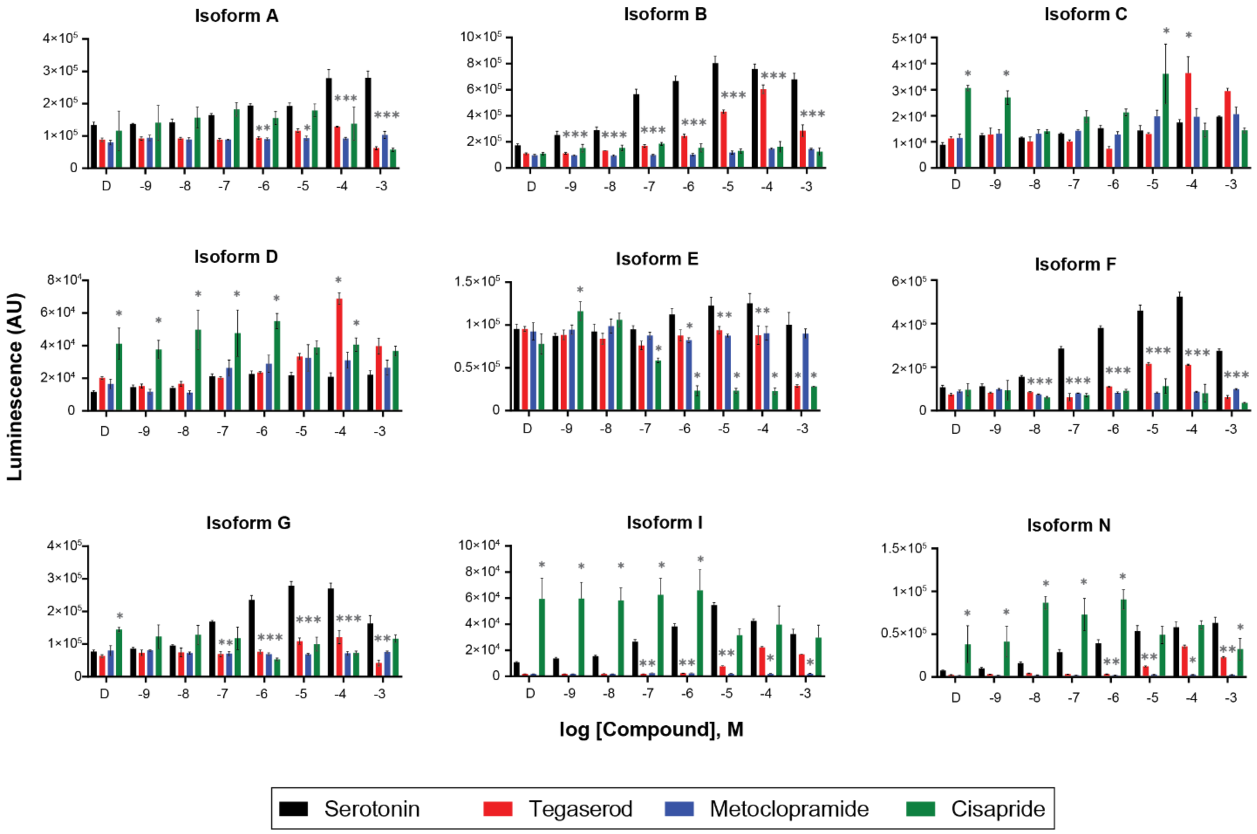

Figure S4. Dose response curves of 5-HTR4 C-terminus isoform-based sensors with serotonin, tegaserod, metoclopramide and cisapride. All experiments were performed in biological triplicates. The bars represent mean ± standard error of mean (SEM), n=3, *p ≤ 0.05. Data were analyzed using two-way ANOVA with Tukey's multiple comparison between signal in the presence of serotonin vs. signal in the presence of other compounds using GraphPad.

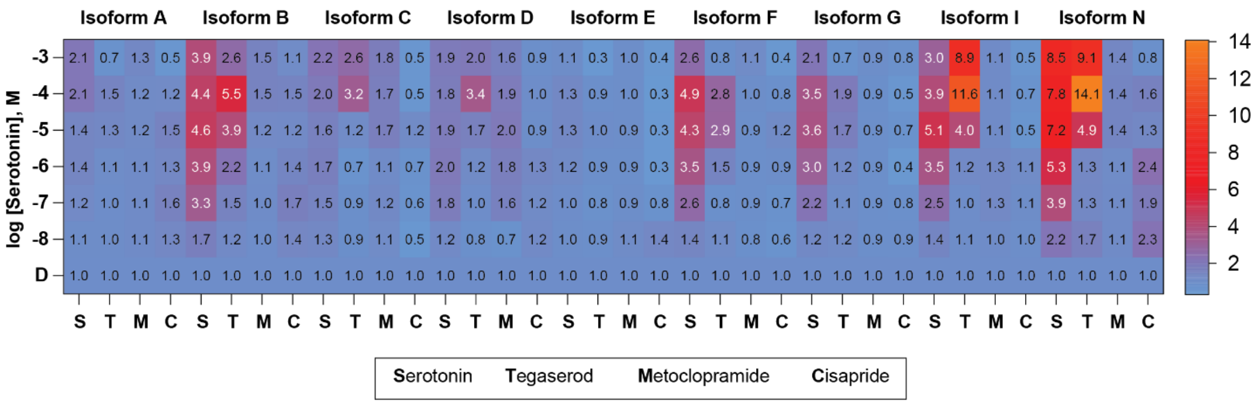

Figure S5. Data on fold increase in signal after activation of single integrated 5-HTR$_4$ C-terminus isoform-based sensor with serotonin (S), tegaserod (T), metoclopramide (M) and cisapride (C).

Sequences

> Human 5-hydroxytryptamine Receptor 4(A) - 5-HTR$_{4A}$ (Q13639-2)

MDKLDANVSSEEGFGSVEKVVLLTFLSTVILMAILGNLLVMVAVCWDRQLRKIKTNYFIVSLAFA
DLLVSVLVMPFGAIELVQDIWIYGEVFCLVRTSLDVLLTTASIFHLCCISLDRYYAICCQPLVYRNK
MTPLRIALMLGGCWVIPTFISFLPIMQGWNNIGIIDLIEKRKFNQNSNSTYCVFMVNKPYAITCSV
VAFYIPFLLMVLAYYRIYVTAKEHAHQIQMLQRAGASSESRPQSADQHSTHRMRTETKAAKTLC
IIMGCFCLCWAPFFVTNIVDPFIDYTVPGQVWTAFLWLGYINSGLNPFLYAFLNKSFRRAFLIILC
CDDERYRRPSILGQTVPCSTTTINGSTHVLRYTVLHRGHHQELEKLPIHNDPESLESCF

> Human 5-hydroxytryptamine Receptor 4(B) - 5-HTR$_{4B}$ (Q13639-1)

MDKLDANVSSEEGFGSVEKVVLLTFLSTVILMAILGNLLVMVAVCWDRQLRKIKTNYFIVSLAFA
DLLVSVLVMPFGAIELVQDIWIYGEVFCLVRTSLDVLLTTASIFHLCCISLDRYYAICCQPLVYRNK
MTPLRIALMLGGCWVIPTFISFLPIMQGWNNIGIIDLIEKRKFNQNSNSTYCVFMVNKPYAITCSV
VAFYIPFLLMVLAYYRIYVTAKEHAHQIQMLQRAGASSESRPQSADQHSTHRMRTETKAAKTLC
IIMGCFCLCWAPFFVTNIVDPFIDYTVPGQVWTAFLWLGYINSGLNPFLYAFLNKSFRRAFLIILC
CDDERYRRPSILGQTVPCSTTTINGSTHVLRDAVECGGQWESQCHPPATSPLVAAQPSDT

> Human 5-hydroxytryptamine Receptor 4(C) - 5-HTR$_{4C}$ (Q13639-9)

MDKLDANVSSEEGFGSVEKVVLLTFLSTVILMAILGNLLVMVAVCWDRQLRKIKTNYFIVSLAFA
DLLVSVLVMPFGAIELVQDIWIYGEVFCLVRTSLDVLLTTASIFHLCCISLDRYYAICCQPLVYRNK
MTPLRIALMLGGCWVIPTFISFLPIMQGWNNIGIIDLIEKRKFNQNSNSTYCVFMVNKPYAITCSV
VAFYIPFLLMVLAYYRIYVTAKEHAHQIQMLQRAGASSESRPQSADQHSTHRMRTETKAAKTLC
IIMGCFCLCWAPFFVTNIVDPFIDYTVPGQVWTAFLWLGYINSGLNPFLYAFLNKSFRRAFLIILC
CDDERYRRPSILGQTVPCSTTTINGSTHVLSSGTETDRKKLWNKEEKIDQTIQMPKRKRKKKAS
LSYEDLILLGRKSCFREGK

> Human 5-hydroxytryptamine Receptor 4(D) - 5-HTR$_{4D}$ (Q13639-3)

MDKLDANVSSEEGFGSVEKVVLLTFLSTVILMAILGNLLVMVAVCWDRQLRKIKTNYFIVSLAFA
DLLVSVLVMPFGAIELVQDIWIYGEVFCLVRTSLDVLLTTASIFHLCCISLDRYYAICCQPLVYRNK
MTPLRIALMLGGCWVIPTFISFLPIMQGWNNIGIIDLIEKRKFNQNSNSTYCVFMVNKPYAITCSV
VAFYIPFLLMVLAYYRIYVTAKEHAHQIQMLQRAGASSESRPQSADQHSTHRMRTETKAAKTLC
IIMGCFCLCWAPFFVTNIVDPFIDYTVPGQVWTAFLWLGYINSGLNPFLYAFLNKSFRRAFLIILC
CDDERYRRPSILGQTVPCSTTTINGSTHVLRF

> Human 5-hydroxytryptamine Receptor 4(E) - 5-HTR$_{4E}$ (X)

MDKLDANVSSEEGFGSVEKVVLLTFLSTVILMAILGNLLVMVAVCWDRQLRKIKTNYFIVSLAFA
DLLVSVLVMPFGAIELVQDIWIYGEVFCLVRTSLDVLLTTASIFHLCCISLDRYYAICCQPLVYRNK
MTPLRIALMLGGCWVIPTFISFLPIMQGWNNIGIIDLIEKRKFNQNSNSTYCVFMVNKPYAITCSV
VAFYIPFLLMVLAYYRIYVTAKEHAHQIQMLQRAGASSESRPQSADQHSTHRMRTETKAAKTLC
IIMGCFCLCWAPFFVTNIVDPFIDYTVPGQVWTAFLWLGYINSGLNPFLYAFLNKSFRRAFLIILC
CDDERYRRPSILGQTVPCSTTTINGSTHVLSFPLLFCNRPVPV

> Human 5-hydroxytryptamine Receptor 4(F) - 5-HTR$_{4F}$ (X)

MDKLDANVSSEEGFGSVEKVVLLTFLSTVILMAILGNLLVMVAVCWDRQLRKIKTNYFIVSLAFA
DLLVSVLVMPFGAIELVQDIWIYGEVFCLVRTSLDVLLTTASIFHLCCISLDRYYAICCQPLVYRNK
MTPLRIALMLGGCWVIPTFISFLPIMQGWNNIGIIDLIEKRKFNQNSNSTYCVFMVNKPYAITCSV
VAFYIPFLLMVLAYYRIYVTAKEHAHQIQMLQRAGASSESRPQSADQHSTHRMRTETKAAKTLC
IIMGCFCLCWAPFFVTNIVDPFIDYTVPGQVWTAFLWLGYINSGLNPFLYAFLNKSFRRAFLIILC
CDDERYRRPSILGQTVPCSTTTINGSTHVLSPVPV

> Human 5-hydroxytryptamine Receptor 4(G) - 5-HTR$_{4G}$ (Q13639-5)

MDKLDANVSSEEGFGSVEKVVLLTFLSTVILMAILGNLLVMVAVCWDRQLRKIKTNYFIVSLAFA
DLLVSVLVMPFGAIELVQDIWIYGEVFCLVRTSLDVLLTTASIFHLCCISLDRYYAICCQPLVYRNK
MTPLRIALMLGGCWVIPTFISFLPIMQGWNNIGIIDLIEKRKFNQNSNSTYCVFMVNKPYAITCSV
VAFYIPFLLMVLAYYRIYVTAKEHAHQIQMLQRAGASSESRPQSADQHSTHRMRTETKAAKTLC
IIMGCFCLCWAPFFVTNIVDPFIDYTVPGQVWTAFLWLGYINSGLNPFLYAFLNKSFRRAFLIILC
CDDERYRRPSILGQTVPCSTTTINGSTHVLSGCSPVSSFLLLFCNRPVPV

> Human 5-hydroxytryptamine Receptor 4(I) - 5-HTR$_{4I}$ (Q13639-8)

MDKLDANVSSEEGFGSVEKVVLLTFLSTVILMAILGNLLVMVAVCWDRQLRKIKTNYFIVSLAFA
DLLVSVLVMPFGAIELVQDIWIYGEVFCLVRTSLDVLLTTASIFHLCCISLDRYYAICCQPLVYRNK
MTPLRIALMLGGCWVIPTFISFLPIMQGWNNIGIIDLIEKRKFNQNSNSTYCVFMVNKPYAITCSV
VAFYIPFLLMVLAYYRIYVTAKEHAHQIQMLQRAGASSESRPQSADQHSTHRMRTETKAAKTLC
IIMGCFCLCWAPFFVTNIVDPFIDYTVPGQVWTAFLWLGYINSGLNPFLYAFLNKSFRRAFLIILC
CDDERYRRPSILGQTVPCSTTTINGSTHVLRTDFLFDRDILARYWTKPARAGPFSGTLSIRCLTA
RKPVLGDAVECGGQWESQCHPPATSPLVAAQPSDT

> Human 5-hydroxytryptamine Receptor 4(N) - 5-HTR$_{4N}$ (Q13639-7)

MDKLDANVSSEEGFGSVEKVVLLTFLSTVILMAILGNLLVMVAVCWDRQLRKIKTNYFIVSLAFA
DLLVSVLVMPFGAIELVQDIWIYGEVFCLVRTSLDVLLTTASIFHLCCISLDRYYAICCQPLVYRNK
MTPLRIALMLGGCWVIPTFISFLPIMQGWNNIGIIDLIEKRKFNQNSNSTYCVFMVNKPYAITCSV
VAFYIPFLLMVLAYYRIYVTAKEHAHQIQMLQRAGASSESRPQSADQHSTHRMRTETKAAKTLC
IIMGCFCLCWAPFFVTNIVDPFIDYTVPGQVWTAFLWLGYINSGLNPFLYAFLNKSFRRAFLIILC
CDDERYRRPSILGQTVPCSTTTINGSTHVLR

References

1. Mukherjee, K.; Bhattacharyya, S.; Peralta-Yahya, P., GPCR-Based Chemical Biosensors for Medium-Chain Fatty Acids. ACS Synth Biol **2015**, 4 (12), 1261-9.
2. Ehrenworth, A. M.; Claiborne, T.; Peralta-Yahya, P. Medium-Throughput Screen of Microbially Produced Serotonin via a G-Protein-Coupled Receptor-Based Sensor. *Biochemistry* **2017,** *56*, 5471-5475.
3. Yasi, E. A.; Allen, A. A.; Sugianto, W.; Peralta-Yahya, P. Identification of Three Antimicrobials Activating Serotonin Receptor 4 in Colon Cells. *ACS Synth Biol* **2019,** *8*, 2710-2717.
4. Zalatan, J. S.; Lee, M. E.; Almeida, R.; Gilbert, L. A.; Whitehead, E. H.; La Russa, M.; Tsai, J. C.' Weissman, J. S.; Dueber, J. E.; Qi, L.S.; Lim, W. A. Engineering complex synthetic transcriptional programs with CRISPR RNA scaffold. *Cell* **2015**, 160, 339-350.
5. Cunningham-Byant, D.; Sun, J.; Fernandez B. Zalatan J.G. CRISPR-Cas-mediated chemical control of transcriptional dynamics in yeast, *ChemBiochem* **2019**, 20, 1519-1523.
6. Brown, A. J.; Dyos, S. L.; Whiteway, M. S.; White, J. H.; Watson, M. A.; Marzioch, M.; Clare, J. J.; Cousens, D. J.; Paddon, C.; Plumpton, C.; Romanos, M. A.; Dowell, S. J. Functional coupling of mammalian receptors to the yeast mating pathway using novel yeast/mammalian G protein alpha-subunit chimeras. *Yeast* **2000,** 16, 11-22.
7. Nakamura, Y.; Ishii, J.; Kondo, A. Applications of yeast-based signaling sensor for characterization of antagonist and analysis of site-directed mutants of the human serotonin 1A receptor. *Biotechnol Bioeng* **2015,** *112*, 1906-15.
8. Lengger, B.; Hoch-Schneider, E. E.; Jensen, C. N.; Jakociu Nas, T.; Petersen, A. A.; Frimurer, T. M.; Jensen, E. D.; Jensen, M. K. Serotonin G Protein-Coupled Receptor-Based Biosensing Modalities in Yeast. *ACS Sensors* **2022,** *7*, 1323-1335.
